# Regulation of the calcium-sensing receptor expression by 1,25-dihydroxyvitamin D_3_, interleukin-6, and tumor necrosis factor alpha in colon cancer cells^☆^

**DOI:** 10.1016/j.jsbmb.2013.10.015

**Published:** 2014-10

**Authors:** Irfete S. Fetahu, Doris M. Hummel, Teresa Manhardt, Abhishek Aggarwal, Sabina Baumgartner-Parzer, Enikő Kállay

**Affiliations:** a Department of Pathophysiology and Allergy Research, Medical University of Vienna, Währinger Gürtel 18-20, Vienna, Austria; b Department of Internal Medicine III, Medical University of Vienna, Währinger Gürtel 18-20, Vienna, Austria

**Keywords:** Calcium-sensing receptor, Colon cancer, Tumor necrosis factor alpha, Interleukin-6, 1,25-dihydroxyvitamin D_3_, Inflammation

## Abstract

Anti-proliferative effects of calcium in the colon are mediated, at least in part, via the calcium-sensing receptor (CaSR), a vitamin D target gene. The expression of CaSR decreases during colorectal tumor progression and the mechanisms regulating its expression are poorly understood. The CaSR promoter harbors vitamin D elements responsive to 1,25-dihydroxyvitamin D_3_ (1,25D_3_) and NF-κB, STAT, and SP1 binding sites accounting for responsiveness to proinflammatory cytokines. Therefore, in the current study we investigated the impact of 1,25D_3_, tumor necrosis factor alpha (TNFα), and interleukin (IL)-6 on CaSR expression in a differentiated (Caco2/AQ) and in a moderately differentiated (Coga1A) colon cancer cell line. 1,25D_3_ induced CaSR expression in both cell lines. Treatment with TNFα was accompanied by a 134-fold induction of CaSR in Coga1A (*p* < 0.01). In Caco2/AQ cells the expression of CaSR was upregulated also by IL-6 (3.5-fold). Our data demonstrated transcriptional and translational activation of the CaSR by 1,25D_3_, TNFα, and IL-6 in a time- and cell line-dependent manner.

This article is part of a Special Issue entitled ‘16th Vitamin D Workshop’.

## Highlights

• 1,25 Dihydroxyvitamin D_3_ induces the expression of CaSR in Caco2/AQ and Coga1A cells.
• TNFα is the main driver of CaSR expression in Coga1A.
• In Caco2/AQ cells 1,25 dihydroxyvitamin D_3_ counteracts the action of TNFα and IL-6.

## 1 Introduction

**Fig. 1:**
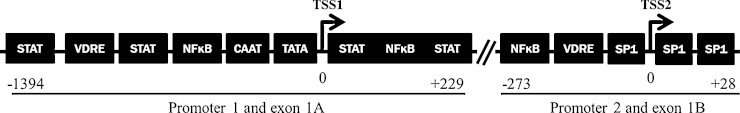
Schematic illustration of the CaSR promoter region including exon 1A and exon 1B. Position of binding sites for regulatory elements is shown (signal transducer and activator of transcription (STAT), vitamin D response elements (VDRE), nuclear factor kappa B (NF-κB), specificity protein 1 (SP1)), which are critical for 1,25D_3_, TNFα, and IL-6 responsiveness, as well as the CAAT and TATA boxes. Transcription start sites (TSS) 1 and 2 according to [12] were taken as point of reference for positioning the indicated binding sites in the corresponding promoters.

Epidemiological studies demonstrate an inverse correlation between calcium and vitamin D intake and risk of tumor development [1], [2]. The calcium-sensing receptor (CaSR) is a putative tumor suppressor gene in the colon, which partially mediates the anti-proliferative and pro-differentiating actions of calcium in colonocytes (for review, see [3], [4]). However, in colon cancer anti-proliferative effects of Ca^2+^ are lost [5], [6], and this could be due to loss of CaSR expression during colorectal tumorigenesis [7]. Very little is known about the factors that regulate the expression of CaSR in the colon. The *CaSR* gene contains 6 coding exons and two 5′-untranslated exons (exons 1A and 1B), which are under the control of promoter 1 and 2, respectively, yielding alternative transcripts but coding for the same protein [8], [9]. Several studies performed in rat parathyroid, thyroid, and kidney have mapped binding sites of numerous transcription factors, including NF-κB, STAT, SP1, and vitamin D response elements in both CaSR promoters (Fig. 1) [9], [10], [11], [12]. Currently, there is limited knowledge regarding the role of 1,25D_3_ and of the proinflammatory cytokines TNFα and IL-6 on CaSR expression in the colon. Therefore, in the present study, we studied the impact of 1,25D_3_, TNFα, and IL-6 on transcriptional and translational regulation of CaSR in two colon cancer cell lines with different proliferation and differentiation properties, mimicking different tumor stages.

## 2 Materials and methods

### 2.1 Cell culture

Caco2/AQ cells are a subclone of the Caco-2 cell line [13]. These carry a truncated APC and a missense mutation of β-catenin, and are able to differentiate spontaneously in culture. In the current study we used highly differentiated, 2 weeks post-confluent Caco2/AQ cells. Coga1A is a cell line derived from a moderately differentiated (G2) colon tumor [14]. These cells are heterozygous for truncated APC, without any known β-catenin mutations [15]. Confluent Caco2/AQ and Coga1A cells were treated for 6, 12, 24, and 48 h either with 10 nM 1,25D_3_, 50 ng/mL TNFα (Sigma Aldrich, USA), 100 ng/mL IL-6 (Immunotools, Germany), or the combination of these compounds. Vehicle treated cells were used as controls.

### 2.2 RNA isolation, reverse transcription, and real time qRT-PCR

RNA isolation and reverse transcription were performed as described previously [16]. Real time qRT-PCR analyses were performed in StepOne Plus system using POWER SYBR GREEN Mastermix following the manufacturer's recommendations (Life Technologies, USA). Data were normalized to the expression of the reference genes: β2M or RPLP0 [17], [18], and set relative to the calibrator (Clontech, USA) to calculate the ΔΔ*C*_T_ value. Primer sequences for CaSR were: 5′-AGCCCAGATGCAAGCAGAAGG-3′ forward, 5′-TCTGGTGCGTAGAATTCCTGTGG-3′ reverse.

### 2.3 Immunofluorescent staining of colon cancer cells

Cells were grown on sterile glass cover slips. After treatments cells were fixed with 3.7% paraformaldehyde in PBS, permeabilized with 0.2% Triton-X (Sigma Aldrich, USA) for 20 min, and blocked with 5% goat serum (Jackson ImmunoResearch, USA). Cells were incubated either with rabbit polyclonal anti-CaSR antibody (1:100, Anaspec, USA) or mouse monoclonal anti-CaSR antibody (1:200, Abcam, UK) for 1 h at room temperature. As negative control we used rabbit or mouse IgG, respectively (Abcam, UK and Life Technologies, USA). As secondary antibody we used Dylight labeled 549 goat-anti-rabbit or Alexa Fluor 647 goat-anti-mouse IgG (1:500, Vector Laboratories and Life Technologies, USA). Nuclei were stained with DAPI (Roche, Switzerland). Images were acquired using TissueFAXS 2.04 (TissueGnostics, Austria).

### 2.4 Statistical analysis

All statistical analyses were performed with SPSS version 18 and graphs were drawn with GraphPad Prism version 5. In case of non-normal distribution, data were log transformed to achieve normal distribution and then subjected to one way ANOVA, followed by Tukey's multiple comparisons posttest. *p*-values smaller than 0.05 were regarded as statistically significant.

## 3 Results

### 3.1 Impact of 1,25D_3_ on CaSR expression

**Fig. 2:**
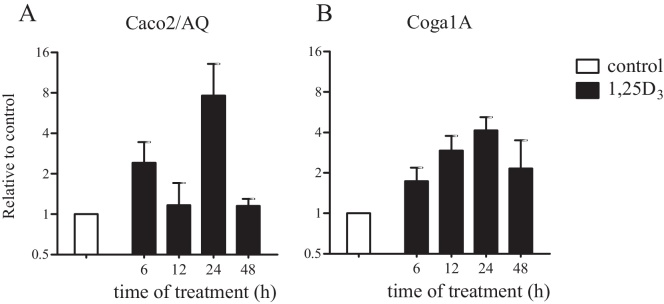
Transcriptional regulation of CaSR by 1,25D_3_ in colon cancer cell lines. Caco2/AQ and Coga1A cells were treated with 10 nM 1,25D3 for the indicated time points. Bars represent mean ± SEM of 2-3 independent experiments.

**Fig. 3:**
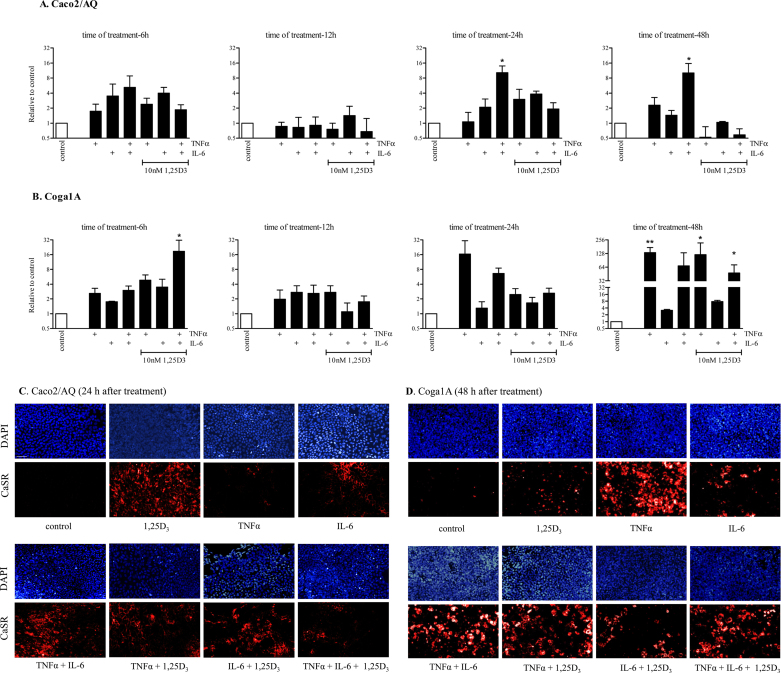
Effect of 1,25D_3_, TNFα, and IL-6 on CaSR expression. (A and B) mRNA expression of Caco2/AQ and Coga1A cells assessed by real time qRT-PCR. Data were log transformed to achieve normal distribution, then subjected to one way ANOVA and corrected with Tukey's posttest for multiple comparisons. Bars represent mean ± SEM of 2-3 independent experiments, asterisks above bars indicate statistically significant changes compared with control. *p < 0.05, **p < 0.01. (C and D) Immunofluorescence staining of the CaSR protein (red) and nuclear staining (blue). Scale bar was 50 μm.

To study the role of vitamin D response elements on transcriptional regulation of CaSR expression we treated Caco2/AQ and Coga1A cells with 1,25D_3_ for 6, 12, 24, and 48 h. In differentiated Caco2/AQ cells treatment with 1,25D_3_ caused 2.4-fold induction of CaSR expression after 6 h. The maximal effect of 1,25D_3_ on CaSR transcriptional activation in these cells was observed at 24 h (7.6-fold; Fig. 2, Fig. 3). In the less differentiated cells Coga1A 1,25D_3_-induced CaSR transcription was 2.9-fold after 12 h and 4.2-fold after 24 h compared with the control group (Fig. 2B). 1,25D_3_ increased CaSR translation as well. Immunofluorescence staining demonstrated upregulation of the CaSR protein in Caco2/AQ after 24 h and Coga1A after 48 h (Fig. 3C and D).

### 3.2 Impact of TNFα and IL-6 on CaSR expression

We treated Caco2/AQ and Coga1A cells with TNFα and IL-6 for 6, 12, 24, and 48 h. In Caco2/AQ treatment with the proinflammatory cytokine TNFα caused only modest upregulation of CaSR expression. Treatment with IL-6 was accompanied by a 3.5-fold induction after 6 h compared with control. Combined treatment with TNFα and IL-6 induced CaSR mRNA expression in Caco2/AQ 10.3-fold (*p* < 0.05) after 24 h and 10.2-fold (*p* < 0.05) after 48 h. However, the combination of all three compounds either had no effect or reduced CaSR expression (Fig. 3A).

In Coga1A cells, treatment with TNFα induced CaSR robustly, especially at 48 h (134-fold, *p* < 0.01). Treatment with IL-6 caused only marginal increases in CaSR mRNA expression. Furthermore, we observed upregulation of CaSR expression in the groups treated with TNFα/IL-6 (68.5-fold) and TNFα/1,25D_3_ (121.2-fold, *p* < 0.05) at 48 h. Similar results were observed in the groups that were treated with TNFα/IL-6/1,25D_3_ at 6 and 48 h (18.8-fold, *p* < 0.05 and 47.7-fold, *p* < 0.05; Fig. 3B).

To address the question whether alterations on CaSR mRNA expression were translated into protein, we performed immunofluorescence staining. Fig. 3C and D demonstrates the upregulation of the CaSR protein upon treatments with the proinflammatory cytokines using the rabbit polyclonal anti-CaSR antibody. Protein expression data were confirmed using the mouse monoclonal anti-CaSR antibody (data not shown). Both antibodies gave the same results.

## 4 Discussion

Recent studies have demonstrated that murine CaSR activates the NLPR3 inflammasome, which in turn induces maturation and release of the inflammatory cytokine interleukin 1β, amplifying the inflammatory signal [19], [20]. Inversely, mice double knockout for CaSR^−/−^/PTH^−/−^ had increased inflammatory response after administration of dextran sodium sulfate compared with control mice expressing the receptor [21]. This suggests an important role for the CaSR in inflammation. Therefore, it is essential to understand how the expression of the CaSR is modulated in the colon.

It has been demonstrated previously that activation of VDREs by 1,25D_3_ and translocation of NF-κB to the nucleus after the treatment with interleukin 1β led to induction of CaSR expression in rat parathyroid, thyroid, and kidneys [9], [10]. Furthermore, IL-6 injection in rats caused induction of CaSR transcription *via* Stat1/3 response elements in promoter 1 and Sp1/3 sites in promoter 2 [11], but not much is known about the regulation of CaSR expression in the colon.

Our study is the first to show that in colonocytes inflammatory cytokines are able to upregulate CaSR expression, and that this effect is time- and cell line-specific. In the present study, we investigated the role of 1,25D_3_, TNFα, and IL-6 on the transcriptional and translational activation of the CaSR in two cell lines representing a highly differentiated and a moderately differentiated colorectal tumor.

1,25D_3_ is known for its anti-proliferative, pro-differentiating effects (for review, see [22]), and its involvement in regulating epigenetic mechanisms [23]. Inducing expression of CaSR, a putative tumor suppressor in the colon, might be one of the tumor preventive mechanisms of 1,25D_3_. In the differentiated Caco2/AQ cells 1,25D_3_ had more pronounced impact in inducing the expression of CaSR than in the less differentiated Coga1A cells. In Caco2/AQ cells treatment with 1,25D_3_ reduced the expression of several proliferation markers also. This was much less evident in the Coga1A cells (data not shown), although the level of the vitamin D receptor is similar [15].

In Caco2/AQ cells, both TNFα and IL-6 increased CaSR expression to a lesser extent than 1,25D_3_. In combination, however, they caused a strong upregulation at 6 h, which was lost at 12 h; at 24 h the effect became additive and the CaSR level remained high also after 48 h. We hypothesized that two different mechanisms were responsible: first, direct upregulation of CaSR expression due to a transient activation of CaSR promoters by NF-κB upon treatment with TNFα and Stat1/3 and Sp1/3 elements by IL-6. This was followed by a second induction of transcription that seems to be indirect. Some (still unknown) factors induced by TNFα and IL-6 might be needed for this more stable induction of CaSR expression. Unexpectedly, 1,25D_3_ counteracted this additive effect, suggesting the existence of intricate feedback systems.

In Coga1A cells, the CaSR was more sensitive to the proinflammatory cytokine TNFα, which was the main driver of CaSR expression in these cells. The low effectiveness of IL-6 in upregulating CaSR expression could be due to lower levels of the IL-6 receptor complex (both the IL-6 binding α chain and the signal transducing unit gp130) in Coga1A cells compared with Caco2/AQ [24]. Interestingly, in these cells the CaSR protein levels remained enhanced in all combined treatments. The robust increase of CaSR expression by TNFα treatment in Coga1A cells could be regarded as a defense mechanism against inflammation. Such protective mechanism was shown in murine macrophages, where lipopolysaccharide-induced TNFα release upregulated CaSR expression leading to inhibition of TNFα synthesis, in a negative feedback manner [25].

In conclusion, our results demonstrate for the first time that in colon cancer cells not only 1,25D_3_, but also the proinflammatory cytokines TNFα and IL-6 were able to induce the expression of CaSR. How this observation can be translated *in vivo* and used for the treatment of inflammation in the gut, still needs to be explored.
